# Compositional Traits of Grains and Groats of Barley, Oat and Spelt Grown at Organic and Conventional Fields

**DOI:** 10.3390/foods12051054

**Published:** 2023-03-01

**Authors:** Lovro Sinkovič, Marianna Rakszegi, Barbara Pipan, Vladimir Meglič

**Affiliations:** 1 Crop Science Department, Agricultural Institute of Slovenia, Hacquetocva ulica 17, SI-1000 Ljubljana, Slovenia; 2 Cereal Breeding Department, Agricultural Institute, Centre for Agricultural Research, Brunszvik u. 2, 2462 Martonvásár, Hungary

**Keywords:** barley, β-glucan, caloric value, farming system, groats, hulling, oat, spelt

## Abstract

Barley, oats, or spelt consumed as minimally processed whole grains provide several health benefits, especially when grown under organic field management conditions. Therefore, the effects of organic and conventional farming on the compositional traits (protein, fibre, fat, and ash) of barley, oat, and spelt grains and groats were compared using three winter barley varieties (‘Anemone’, ‘BC Favorit’, and ‘Sandra’), two spring oat varieties (‘Max’ and ‘Noni’), and three spelt varieties (‘Ebners Rotkorn’, ‘Murska bela’, and ‘Ostro’). Groats were produced from harvested grains by a combination of threshing, winnowing, and brushing/polishing. Multitrait analysis showed significant differences between species, field management practices, and fractions, with clear compositional differences between organic and conventional spelt. Barley and oat groats had a higher thousand kernel weight (TKW) and β-glucan, but lower crude fibre, fat, and ash contents than the grains. The composition of the grains of the different species differed significantly for more traits (TKW, fibre, fat, ash, and β-glucan) than that of the groats (TKW and fat), while field management only affected the fibre content of the groats and the TKW, ash, and β-glucan contents of the grains. The TKW, protein, and fat contents of the different species differed significantly under both conventional and organic growing conditions, while the TKW and fibre contents of grains and groats differed under both systems. The caloric value of the final products of barley, oats, and spelt groats ranged from 334–358 kcal/100 g. This information will be useful for not only the processing industry, but also for breeders and farmers, and last, but not least, for consumers.

## 1. Introduction

Cereal products account for nearly half of the daily caloric intake of people worldwide, ranging from 25% in many European countries to 55% in some developing countries [1]. Cereal grains are the most important source of energy in the diet, with high levels of carbohydrates (70–80%), proteins (7.5–15%), and minerals (1.5–3%), and low levels of fat (1–4%) in human diets worldwide [2,3]. The chemical composition of different cereals depends on their genetic background, environmental and agrotechnical factors, and their interactions, which affect the quality of grains [4]. Recently, the demand for organic products has increased as consumers are becoming more concerned about health, environmental safety, the harmfulness of pesticides, nutrients, bioactive compounds, and safe food [5]. As dietary diversification and the consumption of locally grown foods become increasingly important, many major retailers now offer grain products grown according to organic and sustainable agriculture standards [6]. The use of cereal products made from the least processed whole grains possible is increasingly recommended by nutritionists [1]. Cleaned, hulled, and brushed/polished wholegrain products of barley, oat, and spelt are often an ingredient in the preparation of traditional and modern dishes. Such wholegrain products are available as barley groats, barley kasha, pot barley, barley porridge, pearl barley, whole oat groats, whole grain oats, oat rice, spelt rice, spelt groats, spelt kasha, a mixture of three cereals (rice, spelt, and barley), etc. However, there has been a limited number of studies on the compositional characteristics of the minimally processed seeds of cereals, especially those grown under organic conditions.

Barley (*Hordeum vulgare* L.) is widely cultivated and recognized as a nutritious cereal, but only 2% of the global production is used for human nutrition due to its less favourable organoleptic properties [7]. It is one of the richest sources of carbohydrates and its fibre content is high compared with rice, wheat, sorghum, or corn [3]. Anatomically, barley grain consists of the husk (hull and bran; the tissue surrounding the endosperm and accounting for 7–12% of the grain size) and the endosperm and embryo [8]. Among cereals, oats (*Avena sativa* L.) are considered to be particularly rich in proteins (globulins), phenolic compounds, and dietary fibre, especially β-glucan, as well as various vitamins and minerals [4]. In addition, oats are rich in fat and thiamine, while their energy value is higher than that of other cereals [3]. Oat grains consist of the hull (25%), pericarp, testa, and aleurone (9%), starchy endosperm (63%), and embryo (3%) [9]. Spelt (*Triticum spelta* L.) is an ancient hulled wheat that has attracted new interest in recent years because it is a low-input crop suitable for pesticide-free cultivation in organic farming systems [10]. The disadvantages of spelt cultivation are its high susceptibility to lodging, because the plants are much taller compared with wheat, and the additional step of hulling the grains after harvest [11]. Spelt grains are covered by tough glumes, which protect the grains from external influences, but cause difficulties in harvesting and processing. Therefore, the spikelets require additional treatment after spelt harvest in a process commonly referred to as threshing [12].

Barley, oat, and spelt whole grains have excellent nutritional and bioactive properties due to their fractions, bran, and germ, which contain unique health-promoting bioactive constituents, and have a more complex and beneficial nutritional profile than refined grains [13]. Groats are a wholegrain cereal defined as hulled kernels of various cereals containing the cereal germ and the fibre-rich bran portion of the grain, as well as the endosperm [14,15]. Dietary fibre is an important component of a healthy diet, and β-glucans are one of the most important fractions of soluble dietary fibre [16]. Cereal β-glucans exhibit a number of health-promoting properties in addition to their technological advantages, although the understanding of the complex mechanisms underlying their health benefits is still incomplete [17].

The “raw” grains harvested must be cleaned and brushed/polished by a special technological process before they can be used for human consumption. Winnowing, which usually follows threshing, is a process that separates the chaff from the grain and can also be used effectively to clean and remove pests. Grain winnower machines can be used in agriculture for various grains and do not require much technical training as they are easy to operate [18]. The grains pre-cleaned in this way can be further processed by gentle brushing/polishing, which removes dust, spores, and fungal and dirt particles adhering to the grains without affecting their germination capacity. During polishing, some parts of the seed are lost, which changes the compositional properties of the grain. The removal of dust and infections from the grain has health benefits; however, some of the bioactive components are also removed. The loss of these components and the compositional traits of the groats have been studied in only a few papers.

Thus, samples of barley, oat, and spelt grown under identical environmental conditions in organic and conventional field trials in the same year and at the same location were studied. The compositional characteristics of the harvested barley, oat, and spelt grains, their minimally processed groats (interspecies vs. intraspecies) suitable for direct human consumption were compared, and the effects of growing practices (conventional vs. organic) were evaluated. Groats were evaluated as a first ready-to-eat end product or as a raw material for further processing into wholemeal, flakes, or other products for their composition and potential health benefits.

## 2. Materials and Methods

### 2.1. Plant Materials

A total of eight cereal varieties belonging to three species grown in conventional and organic farming systems were used in this study (Table 1). The three winter barley (*Hordeum vulgare* L.) varieties ‘Anemone’, ‘BC Favorit’, and ‘Sandra’, the two spring oat (*Avena sativa* L.) varieties ‘Max’ and ‘Noni’, and the three winter spelt (*Triticum spelta* L.) varieties ‘Ebners Rotkorn’, ‘Murska bela’, and ‘Ostro’ were grown according to the conventional and organic field management systems established for each species. All cereal varieties were grown in the experimental fields of the Infrastructural Centre Jablje at the Agricultural Institute of Slovenia (46°30′17.4″ N, 15°37′34.6″ E; 320 m a.s.l., subalpine climate) during the winter and/or spring growing seasons of 2017–2018. After harvest, the raw barley, oat, and spelt grains were air-dried before further processing.

### 2.2. Processing of Harvested Grain

**Figure 1:**
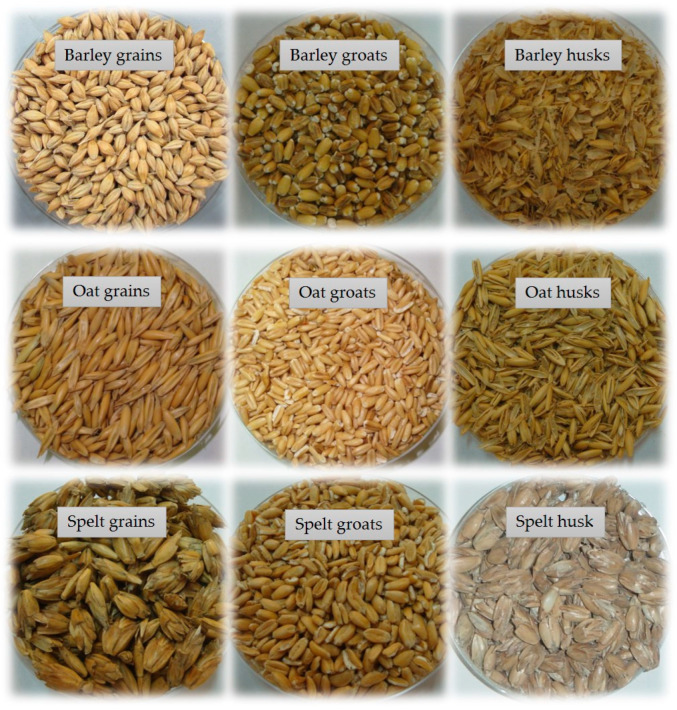
Harvested barley, oat and spelt grains (**left**), minimally processed groats (**centre**) and their husks (**right**).

The harvested and air-dried barley and oat grains were cleaned with a grain winnowing machine with a capacity of 100 kg/h. The harvested spelt grains were threshed with a Wintersteiger LD350 (Wintersteiger AB, Arnstadt, Germany), while a Haldrup DC-20 densimetric column (Haldrup GmbH, Ilshofen, Germany) was used for cleaning. Pre-cleaned grains with husks (barley, oats) or hulled grains (spelt) were further processed by brushing/polishing (barley 3×, oats 7×, and spelt 1×) based on centrifugal force using stone mill equipment specifically designed for processing cereal grains (Table 1). This gentle brushing polished the grains and removed dust, spores, and fungal and dirt particles adhering to the cereal grains without affecting their germination capacity. The final products obtained were called barley, oat, and spelt groats, from which little was removed during processing and which contained all three parts of the grain, i.e., bran, endosperm, and germ. The yield of groats reached about 80% for barley and about 60% for oats and spelt. The raw harvested barley, oat, and spelt grains, the minimally processed groats, and their husks are shown in Figure 1. The thousand kernel weight (TKW) of the harvested and minimally processed barley and oat grains and threshed spelt grains was determined using the Marvin system (MarviTech GmbH, Wittenburg, Germany). The grains and groats of barley, oats, and spelt were homogenized in a laboratory ball mill (Retsch MM400) before further compositional analysis.

### 2.3. Compositional Characteristics and Caloric Value

The chemical composition was determined for all homogenized samples, i.e., determination of dry matter, crude fat, protein, ash, and crude fibre contents, and calculation of moisture, total carbohydrate content, and caloric value. The dry matter content was determined by drying the samples at 103 °C for 48 h (EC 152/2009 App. III A). The Kjeldahl method (ISO 5983:2, 2009) with a factor of 6.25 was used to determine the crude protein content. Crude fats were analysed by petroleum ether extraction (152/2009 App. III H). Five grams of the homogenized sample were placed in an extraction thimble in an extractor and extracted with light petroleum for 6 h. The petroleum extract was collected in a dry, pre-weighed flask. The solvent was distilled and the residue was dried in a drying oven to constant weight. The ash content was determined by the weight difference before and after combustion at 550 °C for 4 h (ISO 5984). The crude fibre content was determined according to ISO 6865:2000. The chemical composition results are expressed as the percentage on a dry matter (DM) basis. The total carbohydrate content was calculated using the following equation: Total carbohydrates = 100—(% protein + % crude fat + % ash + % moisture) [19]. The caloric value (kcal/g) of the groats was calculated using the calorie conversion factors for proteins (4 kcal/g), fats (9 kcal/g), and carbohydrates (4 kcal/g) [20]. The amount of mixed linkage β-glucan was measured in the homogenized samples by enzymatic digestion and spectrophotometry (Evolution 60S, Thermo Fisher Scientific, Waltham, MA, USA) according to AACC 32–23.01. The measurements were performed in two replicates and the results are expressed in mg/g DM.

### 2.4. Statistical Analysis

Least significant differences (LSD) and correlations were calculated using Microsoft Excel 2013 software (Microsoft Corporation, Redmond, WA, USA). Basic statistics included the means, standard deviation (SD), minimum (min), maximum (max), and coefficient of variation (CV). Principal component analysis (PCA) construction was performed in Statistica 6.0 (TIBCO Software Inc., Palo Alto, Santa Clara, CA, USA) to identify the parameters that could discriminate between the different cereal varieties, field management practices (organic vs. conventional farming), and grain fractions (grains vs. groats).

## 3. Results

**Figure 2:**
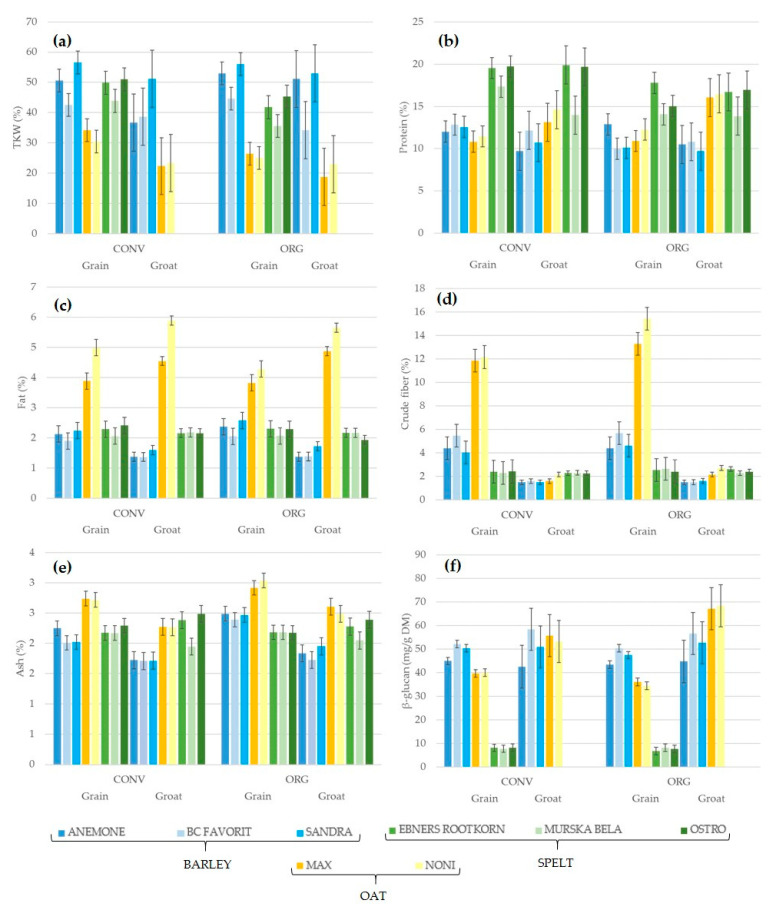
Compositional characteristics of grains and groats of barley, oat, and spelt varieties from conventional (CONV) and organic (ORG) farming: (**a**) TKW, thousand kernel weight; (**b**) protein; (**c**) fat; (**d**) crude fibre; (**e**) ash; and (**f**) β-glucan content. LSD_5%_ values indicate the difference between species/varieties; blue represents barley, orange and yellow represent oat, and green represents spelt varieties.

The compositional characteristics of the harvested barley, oat, and spelt grains and processed groats included the analysis of the TKW, protein, fat, crude fibre, ash, and β-glucan contents (Table 2, Figure 2). The TKW parameter varied considerably from 18.79 g to 56.59 g in the studied cereal samples. The highest mean TKW was found in barley grains (50.58 g), followed by spelt grains (44.58 g) and oat grains (29.04 g), while it was 44.14 g and 21.87 g in barley groats and oat groats, respectively (Table 1). The protein content ranged from 9.70% to 19.81%. The mean protein content was highest in spelt grains (17.26%) and spelt groats (16.85%), and lowest in barley groats (10.60%). The crude fibre content of barley and oats differed significantly between grains and groats, while this difference was very small for spelt. The highest mean crude fibre content was found in oat grains (13.18%) and the lowest in barley groats (1.53%). The fat content ranged from 1.37% in barley groats to 5.24% in oat groats, while the ash content ranged from 1.71% in barley grains to 3.04% in oat groats. The β-glucan content varied significantly among the studied cereals, ranging from 6.74 mg/g DW to 68.40 mg/g DW. The mean β-glucan content was highest in oat groats (61.13 mg/g DM) and lowest in spelt grains (7.78 mg/g DM). The highest coefficients of variation were found for the crude fibre content in oat groats (18.44%), TKW in barley grains (17.56%), and protein content in spelt groats (14.27%).

The differences between the compositional characteristics of barley, oat, and spelt grains and groats according to species/variety from conventional and organic farming are shown in Figure 2. The TKW of grains was significantly higher in the barley and spelt varieties than in the oat varieties (LSD_5%_ = 3.79), while the TKW of groats was significantly higher in the barley varieties compared with the oat varieties (LSD_5%_ = 9.44) (Figure 2a, Table 3) in both field management systems. The protein content of the grains and groats studied did not differ significantly between species/varieties. The fat content of both grains (LSD_5%_ = 0.27) and groats (LSD_5%_ = 0.15) was significantly higher in the oat varieties compared with the barley and spelt varieties (Figure 2c, Table 3). The crude fibre content of the grains was significantly higher in the oat varieties compared with the barley and spelt varieties (LSD_5%_ = 0.97), while the crude fibre content of the groats was not significantly different between species/varieties (Figure 2d, Table 3). The ash content of the grains was significantly higher in the oat varieties than in the barley and spelt varieties (LSD_5%_ = 0.12), while the ash content of the groats was not significantly different between species/varieties (Figure 2e, Table 3). The β-glucan content of the grains was significantly higher in the barley and oat varieties than in the spelt varieties (LSD_5%_ = 0.12). There were no significant differences in the β-glucan content of groats between the barley and oat varieties (Figure 2f, Table 3).

As shown in Table 3, field management had a significant effect on the TKW (LSD_5%_ = 13.76), ash content (LSD_5%_ = 0.43), and β-glucan content (LSD_5%_ = 5.71) of barley, oat and spelt grains. Similarly, it affected the crude fibre content (LSD_5%_ = 0.71) of barley, oat, and spelt groats. Significant differences were found in the TKW (LSD_5%_ = 5.33 and 4.75), protein content (LSD_5%_ = 1.88 and 2.18), and fat content (LSD_5%_ = 0.51 and 0.73) of species and varieties in both management systems (conventional, organic). The TKW and crude fibre content were significantly different between both conventional (LSD_5%_ = 13.04 and 12.79) and organic (LSD_5%_ = 11.61 and 15.16) farming, and the ash content was also significantly different between organic grains and groats (LSD_5%_ = 1.04).

**Figure 3:**
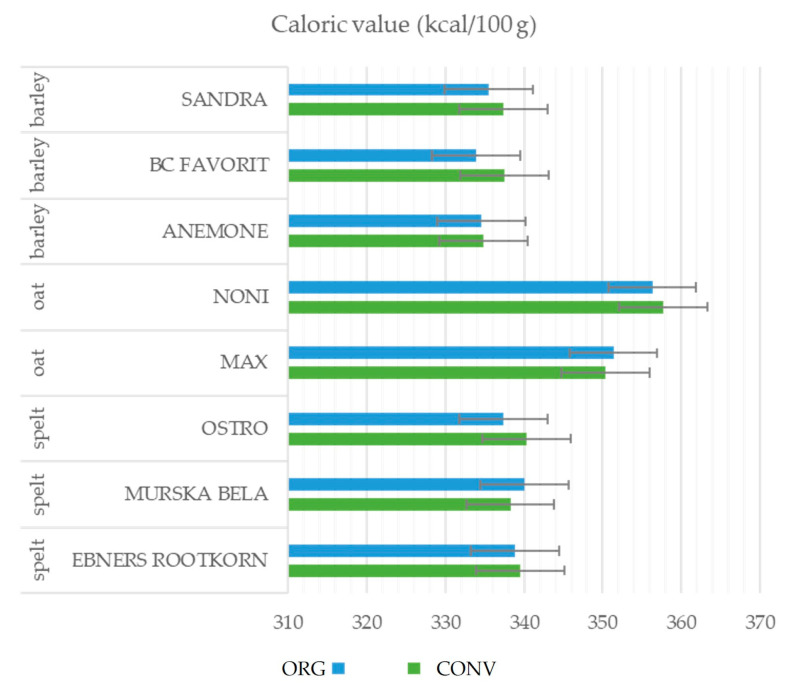
Effects of field management practice (ORG, organic; CONV, conventional) and variety on the caloric value (kcal/100 g) of barley, oat, and spelt groats. LSD_5%_ values showing the differences between the field management practices.

Figure 3 shows the effects of field management (organic vs. conventional) and species/variety on the caloric content of barley, oat, and spelt groats. The highest kcal/100 g was for oat groats of the ‘Noni’ variety grown conventionally (358 kcal/100 g) and the lowest was for barley groats of the ‘BC Favorit’ variety grown organically (334 kcal/100 g). The calorie content of the groats was higher in the oat varieties than in the barley and spelt varieties; however, these differences were not significant.

**Figure 4:**
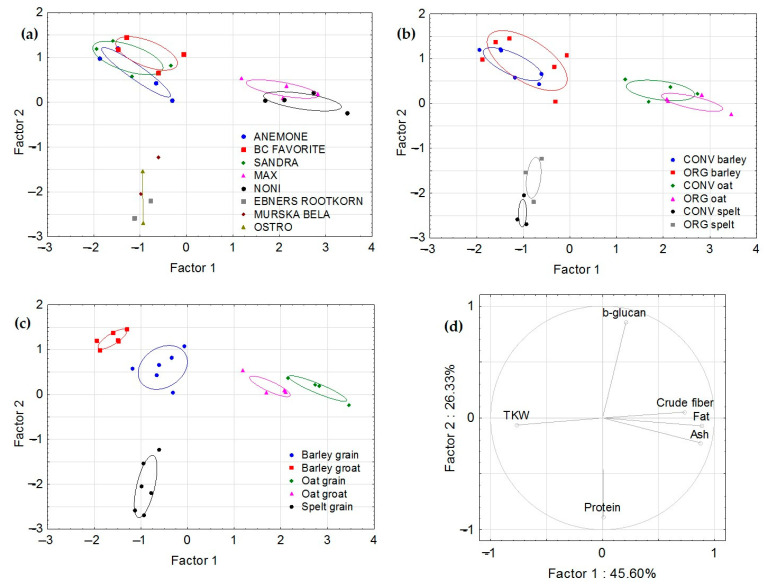
Principal component analysis plots based on six compositional grain characteristics (thousand kernel weight (TKW) and β-glucan, protein, crude fibre, fat, and ash contents) grouped by (**a**) cereal variety, (**b**) field management, and (**c**) grain fractions. (**d**) Discriminant loading plot. Factor 1 is determined by the fat (0.8889), ash (0.8786), and crude fibre (0.7332) contents and TKW (−0.7693), while Factor 2 is determined by the protein (−0.8858) and β-glucan (0.8562) contents.

The statistical analysis of the data obtained was performed using principal component analysis (PCA) to identify the parameters responsible for the discrimination between samples. Samples were grouped on plots in three different ways: by species/variety (barley vs. oats vs. spelt), by field management technique (organic vs. conventional), and by the grain fraction studied (harvested grains vs. minimally processed groats). The results of the PCA analysis are shown in Figure 4 as discriminant function score plots (a, b, and c) and a discriminant loading plot (d). In plots (a, b, and c), the observations and multivariate means of each group (i.e., the centroids) are presented as scatter plots, while in plot (d), the set of vectors is presented as a loading plot indicating the degree of association of the corresponding output parameters with the first two discriminant functions, representing 71.93% of the total variance. The relative magnitude of the standardized discriminant function values for discriminant function 1 (45.60% of the total variance) indicated that fat, ash, TKW and crude fibre were the main factors for this function, in that order.

As shown in Figure 4a–c, three separate groups were formed. The samples of barley varieties (a) are located close to each other in the upper-left part of the plot and the spelt samples are in the lower-left part of the plot. The oat samples are clearly different from those of barley and spelt and are in the right part of the plot. Good separation between cereal samples according to the field management technique can be seen for spelt, while barley and oat samples overlap slightly (b). Good separation between samples by grain fraction is also evident in plot (c) for barley and oat samples. A comparison of the plots in Figure 4 by correlating the positions of the different groups in the left plot with the positions, directions, and lengths of the individual vectors in the right plot reveals the crucial parameters responsible for the separation of the groups. The most influential parameter for discriminating between barley and oat samples in the left part of the plots (a, b, and c) correlates with the vector of TKW in the discriminant loading plot (d) (i.e., with the highest mean values). In contrast, the vectors of crude fibre, fat and ash correlate with the oat samples, which means that these parameters reach their maximum mean values in these groups. Moreover, the higher content of β-glucan distinguishes the barley and oat samples from the spelt samples. Additionally, the higher protein content distinguishes the spelt samples from the barley and oat samples.

## 4. Discussion

The consumption of whole grain products is increasing due to the growing health consciousness of the population, and health-promoting components support the dietary consumption of these products [21]. Among the different cereals, barley (*Hordeum vulgare*), oats (*Avena sativa*), and spelt (*Triticum spelta*) were selected because they are widely used in production and human consumption as groats, flakes, wholemeal, etc. To further explore the uniqueness and health potential of whole grain products, the specific characteristics of the grain of each cereal species and their processing methods need to be investigated [22]. Therefore, the aim of this study was to analyse the nutritional characteristics of the above-mentioned cereals before and after grain processing. Barley, oats, and spelt are small-grain cereals available in the food market in various forms, including minimally processed whole grains and groats, suitable for the preparation of traditional and modern dishes [22,23]. The harvested grains of these cereals must be threshed, cleaned, and/or brushed/polished before they can be used for direct consumption.

In the present study, harvested barley, oat, and spelt grains were used to produce, in a few steps, a minimally processed, ready-to-eat food product that can be used for cooking or for processing into other products, such as wholemeal or flakes. In the case of spelt, the harvested air-dried grains were threshed to dehull them, which was an additional step before further cleaning. The cleaning of the grains, i.e., barley and oat with the winnower machine and spelt with the Haldrup DC-20, was based on the differences in the width, thickness, and length of the grains, as well as their aerodynamic properties, surface texture, shape, and specific gravity. The harvested air-dried barley and oat grains, and the threshed spelt grains were processed in cleaning machines based on previous experience for each type of grain. The next step entailed the brushing/polishing of the pre-cleaned grains using a traditional stone mill. In this process, the grains were rotated in the drum mill different numbers of times (barley 3×, oats 7×, and spelt 1×) to obtain a minimally processed, ready-to-eat whole grain product, called groats, of each respective cereal. After applying these processing steps to the harvested raw grains, the yield of groats reached about 80% for barley and about 60% for oats and spelt. The barley groats obtained in this way generally have various names, such as pearl barley, pearled barley, or pot barley.

From a grain fraction perspective (harvested grains vs. minimally processed groats), the TKW and crude fibre content were generally higher in grains, whereas the content of β-glucans was higher in groats. The protein and fat contents were higher in oat groats, and the ash content was lower in barley and oat groats. From the cereal species/variety point of view (interspecies vs. intraspecies), the TKW of oats was generally lower than that of barley and spelt, the protein content was higher in spelt, the β-glucan contents were lower in spelt, while the fat, crude fibre, and ash contents were higher in oats. The barley variety ‘BC Favorit‘ had a generally lower TKW than ‘Anemone’ and ‘Sandra’, while the crude fibre and β-glucan contents were higher. Among the oat varieties, ʹNoniʹ generally had a higher fat content than ‘Max‘, while the differences between the other parameters were not large. Among the spelt varieties, the TKW and protein content of ‘Murska bela’ were generally lower than those of ‘Ebners Rotkorn‘ and ‘Ostro‘. The TKW is directly related to the grain yield and milling quality of the grain and is closely linked to the grain size characteristics. Therefore, it is widely used in crop research as a measurement indicator that depends on the variety, environmental conditions, and agricultural practices [8,24]. As reported by Hejcman et al. [25], the descending order of the cereals we studied in terms of their TKW relative to *Triticum aestivum* was comparable when the TKW of *T. aestivum* (44.6 g) was taken as 100%, i.e., *Triticum spelta* (100%), hulled *Hordeum vulgare* (99%), and hulled *Avena sativa* (49%). Kulathunga et al. [15] found an average TKW of 38.3 ± 4.9 g in the threshed grains of three spelt genotypes, while Tóth et al. [23] studied 90 spelt genotypes whose TKWs ranged from 23.2–49.7 g and protein contents ranged from 12.1–22.2%. The results regarding the protein, crude fibre, fat, and ash contents in the harvested unprocessed barley and oat grains are in agreement with those of previous reports [2,4,8]. Kulathunga et al. [15] reported slightly lower mean contents of protein (15.2%), fat (1.6%), and ash (2.1%) in spelt grains of three genotypes compared with our spelt data.

The β-glucan content of barley is generally up to 11%, which is higher than that of oats and wheat [26]. The health benefits of whole grain barley and oats are well known, but the mechanisms responsible for them are challenging due to the complexity, structure, chemical composition, and effects of processing grains into foods. In this context, viscous soluble β-glucans have been shown to play an important role in lowering cholesterol and the postprandial blood glucose levels, while dietary fibre and phytonutrients also play a role in maintaining a healthy gut microbiota [27]. The soluble β-glucan content in the studied cereal samples was significantly higher in barley (average 6.2-fold) and oat grains (average 4.8-fold) compared with spelt grains. After processing the harvested grains, barley and oat groats contained even higher levels of β-glucans, which were 6.6-fold and 7.9-fold higher, respectively. This trend is consistent with data from the literature on β-glucans in barley (2–17%), oats (2–9%), and spelt (0.5–0.9%) [16,17,23]. Barley β-glucans are distributed mainly in the endosperm of the grain and, in the case of oats, in the thick cell walls in the subaleuron region of the outer endosperm of the grain [16], which explains the higher levels in oat groats after grain processing. According to the European Food Safety Authority (EFSA), 4 g of β-glucans from oats or barley per 30 g of available carbohydrate should be consumed per meal to reduce the post-prandial glycaemic response [28]. Regular consumption of minimally processed barley and oat groats could help to increase dietary intake of β-glucans.

From a field management perspective (conventional vs. organic), the crude fibre content was generally higher in organic crops, the TKW and fat content were higher only in organic barley, the protein content was higher in conventional barley and spelt, the ash content was higher in organic barley and oats, and the β-glucan content was higher only in organic oats. The cultivation of hulled wheat (*Triticum spelta*) has been associated with the expansion of organic farming and the increasing interest in products with high nutritional quality in the past two decades, although *Hordeum* varieties dominate in today’s Europe [25]. Although organic spelt already occupies a niche market in North America and Europe [15], there are still many opportunities for organic or small-scale farms to offer minimally processed spelt products, such as whole grains and groats. Dolijanović et al. [11] reported significant differences in organic spelt production between different regions, i.e., lowland, hilly, and mountainous. Significantly higher protein contents were reported for spelt than for common wheat, but there were no differences between conventional and organic farming systems [1]. Menkovska et al. [29] reported a higher β-glucan content in conventionally grown barley (34.4 and 33.3 mg/g DM) and oat grains (23.5 and 21.3 mg/g DM) compared with those grown organically. However, the β-glucan contents were significantly higher in barley (42.6–58.4 mg/g DM) and oat grains and groats (34.5–68.4 mg/g DM) in our study. Multivariate analysis (PCA) showed that the three species were well-separated based on their studied compositional characteristics. While oats and barley had high β-glucan contents, with a low TKW of oats, spelt is rich in proteins. Field management had the greatest influence on spelt varieties, but oat samples were also well separated. At the same time, the compositional characteristics of grains and groats were most different between barley varieties, while there was no difference in spelt.

Cereal grains generally have high energy value and consist mainly of starch, but also of protein (6–12%) and fat (1–5%) [30]. As there were no significant differences in the fat and protein contents between conventionally and organically produced grains, comparable caloric values were expected and confirmed. The caloric value of barley, oat, and spelt groats studied was 334–358 kcal/100 g, slightly lower than the caloric value of pearl barley and oat groats (470 kcal/100 g) reported by Beloshapka et al. [31]. The higher caloric value of oats compared with barley was reported previously, at 389 kcal and 350 kcal, respectively [3].

## 5. Conclusions

The compositional characteristics of the studied cereals showed large differences between species, within species, between organic and conventional farming systems, and also between the processed fractions of the grains. The application of threshing, cleaning, and/or brushing/polishing processes to harvested grains resulted in nutritious, ready-to-eat grain products that were suitable for cooking or further processing into flakes or wholemeal. Barley and oat groats grown conventionally or organically had higher β-glucan contents compared with spelt, while spelt had a significantly higher protein content. Our studies demonstrated that polishing had a positive effect on the β-glucan contents of oat and barley, and that both the β-glucan and crude fibre contents were higher in spelt and barley from organic field management than in the conventional system. The consumption of wholegrain cereals and cereal products is recommended for every meal to the dietary guidelines for healthy living. Organic farming and the use of groats could help to increase dietary fibre consumption among consumers.

## Figures and Tables

**Table 1:** List of studied cereal varieties grown in 2017/2018 and technological procedures applied to the harvested grains.

Species	Latin Name	Type	Variety Name	Field Management	Technological Procedures Applied to Harvested Grain
Barley	*Hordeum vulgare* L.	Winter	Anemone	CONV	(i) Cleaning with a grain winnowing machine; (ii) Brushing/polishing (3×) of the cleaned raw grains by centrifugal force with an adapted traditional stone mill.
				ORG	
			BC Favorit	CONV	
				ORG	
			Sandra	CONV	
				ORG	
Oat	*Avena sativa* L.	Spring	Max	CONV	(i) Cleaning with a grain winnowing machine; (ii) Brushing/polishing (7×) of the cleaned raw grains by centrifugal force with an adapted traditional stone mill.
				ORG	
			Noni	CONV	
				ORG	
Spelt	*Triticum spelta* L.	Winter	Ebners Rotkorn	CONV	(i) Threshing with a Wintersteiger LD359 machine; (ii) Cleaning of the hulled raw grains with Haldrup DC-20; (iii) Brushing/polishing (1×) of the cleaned raw grains by centrifugal force with an adapted traditional stone mill.
				ORG	
			Murska bela	CONV	
				ORG	
			Ostro	CONV	
				ORG	

CONV, conventional; ORG, organic.

**Table 2:** The summary data of the compositional characteristics studied in the harvested grains and processed groats of barley, oat, and spelt.

Parameter	Unit	Statistical Parameter	*n*	Barley		*n*	Oat		*n*	Spelt	
				Grains	Groats		Grains	Groats		Grains	Groats
TKW	g	Min-Max	6	42.57-56.59	34.22-52.99	4	25.03-34.22	18.79-23.40	6	35.58-50.98	/
		Mean		50.58	44.14		29.04	21.87		44.58	/
		SD		5.34	7.75		3.60	1.82		5.14	/
		CV (%)		10.56	17.56		12.41	8.32		11.53	/
Protein	%	Min-Max	6	10.00–12.87	9.70–12.17	4	10.83–12.27	13.13–16.48	6	14.07–19.73	13.86–19.81
		Mean		11.73	10.60		11.37	15.07		17.26	16.85
		SD		1.23	0.83		0.57	1.31		2.11	2.40
		CV (%)		10.45	7.84		5.06	8.72		12.23	14.27
Crude fibre	%	Min-Max	6	4.04–5.68	1.49–1.61	4	11.86–15.43	1.59–2.71	6	2.29–2.64	2.26–2.62
		Mean		4.76	1.53		13.18	2.15		2.45	2.35
		SD		0.60	0.05		1.40	0.40		0.11	0.13
		CV (%)		12.63	3.39		10.65	18.44		4.58	5.42
Fat	%	Min-Max	6	1.90–2.58	1.37–1.72	4	3.82–4.99	4.55–5.89	6	2.06–2.41	1.94–2.18
		Mean		2.21	1.47		4.24	5.24		2.24	2.12
		SD		0.22	0.14		0.47	0.55		0.13	0.08
		CV (%)		9.97	9.45		10.99	10.47		5.78	3.97
Ash	%	Min-Max	6	2.01–2.49	1.71–1.95	4	2.72–3.04	2.26–2.61	6	2.17–2.30	1.95–2.49
		Mean		2.27	1.78		2.86	2.41		2.20	2.26
		SD		0.20	0.09		0.13	0.15		0.04	0.19
		CV (%)		8.68	5.04		4.63	6.06		2.01	8.64
β-glucan	mg/g DM	Min–Max	6	43.39–52.18	42.62–58.43	4	34.50–40.07	53.20–68.40	6	6.74–8.22	/
		Mean		48.14	51.02		37.58	61.13		7.78	/
		SD		3.15	5.77		2.35	6.76		0.52	/
		CV (%)		6.55	11.31		6.24	11.05		6.62	/

TKW, thousand kernel weight; DM, dry matter; SD, standard deviation; CV, coefficient of variation.

**Table 3:** Significant differences among the species/varieties and field management practices as shown by the LSD_5%_ values given for different fractions and field management techniques.

LSD Statistic		TKW	Protein	Crude Fibre	Fat	Ash	β-glucan
Grains	Species/variety	3.79	1.26	0.97	0.27	0.12	1.57
	Field management	13.76	4.57	3.51	0.97	0.43	5.71
	Species/variety	***	n.s.	***	***	***	***
	Field management	*	n.s.	n.s.	n.s.	***	*
Groats	Species/variety	9.44	2.25	0.20	0.15	0.14	8.95
	Field management	23.08	8.16	0.71	0.54	0.50	21.89
	Species/variety	*	n.s.	n.s.	***	n.s.	n.s.
	Field management	n.s.	n.s.	*	n.s.	n.s.	n.s.
CONV	Species/variety	5.33	1.88	3.53	0.51	0.24	9.82
	Fraction	13.04	6.84	12.79	1.84	0.88	24.02
	Species/variety	**	**	n.s.	***	n.s.	n.s.
	Fraction	*	n.s.	*	n.s.	n.s.	n.s.
ORG	Species/variety	4.75	2.18	4.18	0.73	0.29	19.38
	Fraction	11.61	7.91	15.16	2.64	1.04	47.41
	Species/variety	***	*	n.s.	**	n.s.	n.s.
	Fraction	*	n.s.	*	n.s.	*	n.s.

n.s., not significant; *, **, *** significant at 0.05, 0.01, and 0.001 probability levels. CONV, conventional; ORG, organic; TKW, thousand kernel weight.

## Data Availability

Data are contained within the article.
